# Personalized Scrub Caps for Identification of Surgical Trainees

**DOI:** 10.1001/jamanetworkopen.2023.32403

**Published:** 2023-09-07

**Authors:** Divyansh Agarwal, Tina Bharani, John T. Mullen

**Affiliations:** 1 Department of Surgery, Massachusetts General Hospital, Boston, Massachusetts; 2 Department of Surgery, Brigham & Women’s Hospital, Boston, Massachusetts

## Abstract

This survey study investigates whether personalized scrub caps for surgical trainees can help decrease role and name misidentification, microaggressions, and miscommunication-related delays in patient care.

## Introduction

In the operating room (OR), communication failures occur in up to 30% of procedurally relevant interactions, jeopardizing patient safety.^1^ An attending surgeon is typically unable to name more than half the staff during an OR procedure.^2^ Surgical trainees constitute a particularly at-risk cohort for being misidentified as nonphysicians, with higher prevalence among those who are female or underrepresented with minoritized race and ethnicity (URM).^3,4^ Physician role and name misidentification have been associated with increased imposter syndrome, communication errors, and burnout,^3,4^ which can reduce the quality of patient care^5^ and lead to adverse patient outcomes.^6^ Interventions to improve role and name identification, such as identity badges, are associated with reduced misidentification and improved work experience among trainees.^4^ However, these are of limited utility in the OR. We examined whether personalized scrub caps can help decrease role and name misidentification, microaggressions, and miscommunication-related delays in patient care.

## Methods

This survey study was exempted by the Massachusetts General Hospital institutional review board because the data were deidentified. All participants provided informed consent. We followed the AAPOR reporting guideline.

**Figure 1.**
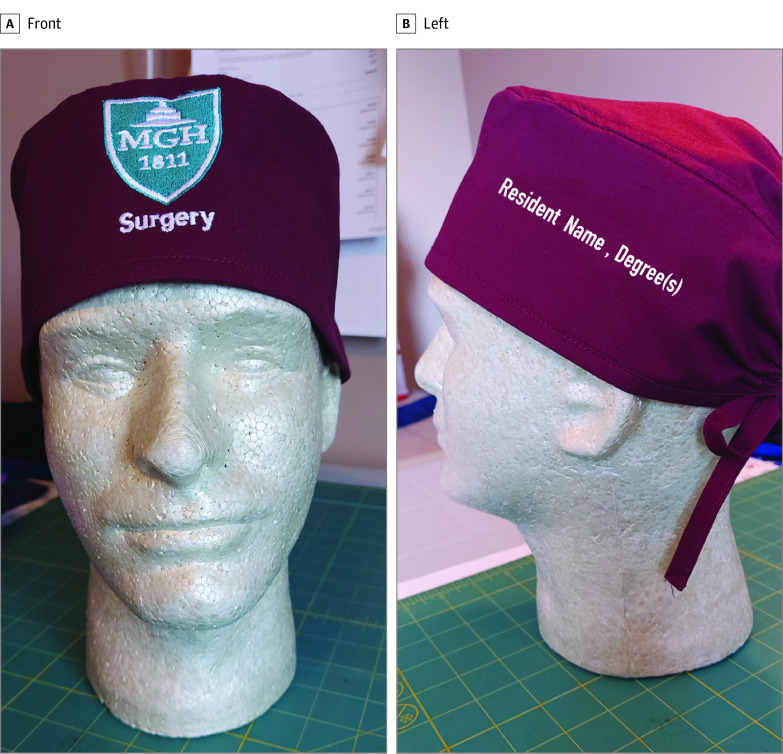
Scrub Cap With Preferred Name Embroidered A scrub cap with preferred name embroidered was provided to all surgery house staff.

Personalized scrub caps (Figure 1) were provided to 92 surgical trainees at the Massachusetts General Hospital. An electronic survey was administered in October 2022 (prior to cap distribution) and 6 months later in April 2023. Survey items were Likert-type scales and trainees were asked to report how often their name and/or role was misidentified, and whether the misidentification resulted in delay in patient care, near-miss clinical events, or patient harm. Summary statistics were used to characterize the responses, stratified by gender, URM, and international medical graduate (IMG) status. Two sample *Z*-tests were used to compare proportions, and the McNemar test was used for paired analysis of trainees experiencing a patient care issue because of name and/or role misidentification before and after cap use. Fisher exact tests were used to identify associations between responses and gender, URM, and/or IMG status. Two-sided *P* < .05 was considered significant. All analyses were done in R version 3.4.0 (R Project for Statistical Computing).

## Results

**Figure 2.**
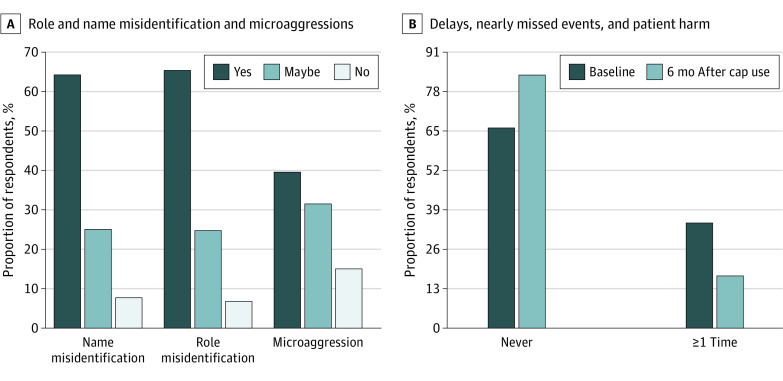
Proportion of Respondents Reporting Role and Name Misidentification, Microaggressions, Delays, Nearly Missed Events, and Patient Harm at Baseline and 6 Months After Cap Use Personalized scrub caps were associated with a decrease in (A) role and name misidentification and microaggressions and (B) the overall proportion of respondents who reported experiencing a delay in patient care, a nearly missed clinical event, or patient harm because of name or role misidentification. A, Question asked of respondents: “Did wearing a personalized scrub cap decrease any of the following?” B, Question asked of respondents: “Over the last 6 months, how often has there been a delay in patient care, a nearly missed clinical event, or patient harm because of misidentification of your name or role?”

Of the 64 surgical trainees (69.6%) who completed both the baseline and 6-month surveys, 34 were female (53.1%) and 30 were male (46.9%); 6 were IMGs. Among the 13 trainees who self-identified as URM and received the caps initially, 12 (92.3%) completed both surveys. Fifty trainees (78.1%) reported wearing the scrub cap multiple days per week. After 6 months of scrub cap use, 42 (65.6%) reported that the caps helped decrease name misidentification, 43 (67.2%) reported a decrease in role misidentification, and 23 (35.9%) described a decrease in microaggressions (Figure 2A). Among female and URM trainees, the proportion of respondents who experienced a decrease in name misidentification, role misidentification, and microaggressions was significantly higher compared with male and non-URM trainees (eg, experienced a reduction in role misidentification: 95.0% of female and URM trainees [38 of 40] vs 20.8% of male and non-URM trainees [5 of 24]; *P* < .001).

We matched the baseline and 6-month surveys for each respondent to compare the proportion of trainees who reported 1 or more patient care errors because of misidentification; this decreased from 34.4% (22 of 64) in the baseline survey to 14.1% (9 of 64) (*P* = .003) in the 6-month survey (Figure 2B). Being a female trainee was associated with a reduction in role misidentification (odds ratio, 1.62 [95% CI, 1.12-3.77]; *P* < .001); being an IMG or URM trainee was associated with a reduction in name misidentification and microaggressions (odds ratio, 2.08 [95% CI, 1.26-5.37]; *P* < .001). Moreover, 42 respondents (65.6%) perceived that personalized scrub caps helped create a more professional perception among the patients they saw. Of note, approximately two-thirds of the participants also reported that wearing the personalized scrub cap improved critical communication in emergency department while running a trauma code.

## Discussion

This study found that scrub caps embroidered with trainee names were associated with reduced role and name misidentification, perceived microaggression, and decrease in frequency of nearly missed events or delays in patient care. The largest difference in reduction of misidentification was evident in female and URM trainees, likely due to the higher prevalence of workplace discrimination experienced by women and physicians with minoritized race and ethnicity. We surmise that personalized scrubs helped reduce microaggressions and possible delays in patient care by reducing interpersonal conflicts that arise from poor communication. Our study’s results suggest practical implications for surgery residency training institutions and residency program leadership. Limitations of this study include this being a single-center nonrandomized study and reliance on memory recall of events. Getting to know trainees over the 6-month study period may have also contributed to reduced misidentification.

## Notes

Supplement.
Data Sharing Statement
