# Supplementary material for: Personalized Scrub Caps for Identification of Surgical Trainees
Source: JAMA Netw Open. 2023 Sep 7;6(9):e2332403. doi: 10.1001/jamanetworkopen.2023.32403 (PMC10485725; doi:10.1001/jamanetworkopen.2023.32403)
Supplement: Supplement. — Data Sharing Statement [file jamanetwopen-e2332403-s001.pdf]

Data Sharing Statement

Agarwal. Personalized Scrub Caps for Identification of Surgical Trainees. *JAMA Netw Open*. Published September 07, 2023. doi:10.1001/jamanetworkopen.2023.32403

Data
Data available: Yes
Data types: Data (not involving human participants)
How to access data: Please email Dr. Agarwal (dagarwal@mgh.harvard.edu) for any data requests.
When available: With publication

Supporting Documents
Document types: None

Additional Information
Who can access the data: Anyone requesting the data
Types of analyses: For any research purpose
Mechanisms of data availability: With investigator support
